# Carbon nanomaterials for phototherapy

**DOI:** 10.1515/nanoph-2022-0574

**Published:** 2022-11-21

**Authors:** Xichu Wang, Lin Zhu, Zi Gu, Liming Dai

**Affiliations:** Australian Carbon Materials Centre (A-CMC), University of New South Wales, Sydney, New South Wales 2052, Australia

**Keywords:** carbon nanomaterial, photodynamic therapy, phototherapy, photothermal therapy, surface modification

## Abstract

Phototherapy attracts increasing interest for broad bio-applications due to its noninvasive and highly selective nature. Owing to their good biocompatibility, unique optoelectronic properties and size/surface effects, carbon nanomaterials show great promise for phototherapy. Various carbon nanomaterials have been demonstrated as efficient phototherapy agents for a large variety of phototherapeutic applications, including cancer treatment, anti-bacteria, and Alzheimer’s disease. This review summarizes the recent progress of carbon nanomaterials for phototherapy. Current challenges and future perspectives are also discussed.

## 1 Introduction

Phototherapy, as minimally invasive therapy, is widely applied for cancer therapy, anti-bacterial/inflammation, and other biomedical applications [1–3]. Compared to chemotherapy or radiotherapy, phototherapy exhibits an extra level of selectivity by focusing light beams on the targeted region to minimize side effects [4–6]. A typical phototherapeutic process involves light sources and phototherapeutic agents (PAs) that can convert light into thermal energy (photothermal therapy, PTT) or chemical energy (photodynamic therapy, PDT). Among the light sources typically used for phototherapy, UV–vis light (200–700 nm) has relatively high energy but limited penetration depth while near-infrared (NIR) light (800–1200 nm) with an approximate penetration depth of 20–30 mm has relatively low energy. NIR light is generally considered a promising excitation source for deep-tissue applications (e.g., deep-seated tumours) [4].

**Figure 1:**
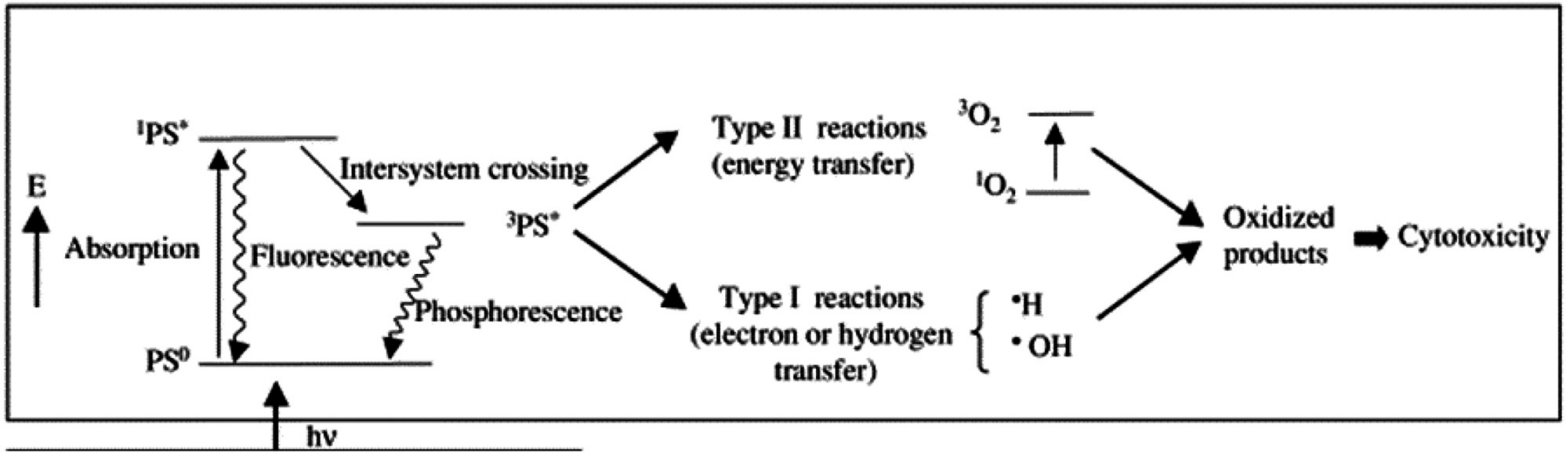
Photodynamic therapy (PDT) mechanism [14]. Copyright 2022 Elsevier publishing group.

Photothermal therapy (PTT) converts the light into heat to induce localized hyperthermia (>39 °C) for ablating the targeted area (e.g., tumour [5–7], bacteria [8–10] through the destruction of cells (e.g., protein denature at 39 °C). However, light-induced hyperthermia often requires a high-power intensity, leading to potential damage to non-target cells [11]. In the process of photodynamic therapy (PDT), the photodynamical agents or photosensitizers [12] (PS) absorb the light of a particular wavelength and transfer the photo energy to surrounding molecules to produce highly reactive oxygen species (ROS) [13], as schematically shown in Figure 1 [14]. Therefore, based on the specific energy transfer process involved, PDT can be further divided into type I, electron or hydrogen transfer to produce radicals (e.g., ·OH, O_2_^·−^) [15]; and type II, energy transfer to produce singlet oxygen [12, 16]. For tumour therapy, type I attracts more and more attention because of its low oxygen dependency and the hypoxia characteristic in the tumour microenvironment (TME) [12].

Although considerable progress in phototherapy has been made recently [17], current phototherapeutic treatments suffer several drawbacks, including high laser intensity, potential skin damage, limited penetration depth, and poor targeting [18–20]. PDT for cancer treatment is further limited by the fact that the hypoxia characteristic in the TME [13] promotes tumour growth, abnormal tumour vasculature, and metastasis with resistance to O_2_ dependence treatments [4, 21, 22] and that PDT agents generally respond to UV-visible lights, which has the poor tissue penetration and a high potential for normal tissue damage due to the relatively high light energy.

**Figure 2:**
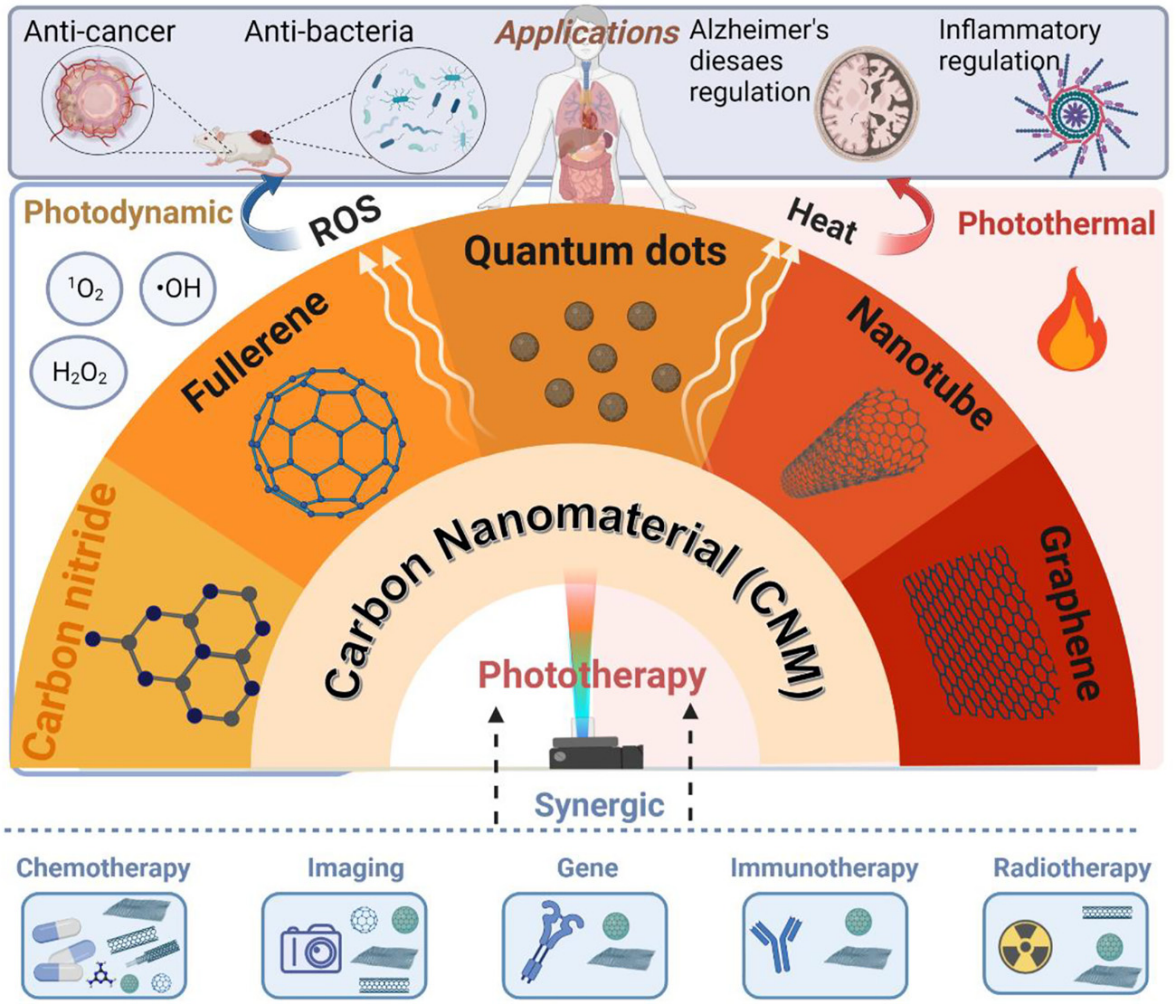
Summary of carbon nanomaterials (CNMs) for phototherapy, created with BioRender.

Currently-used phototherapeutic agents (PAs) include organic molecules, noble metal oxides, and semiconducting nanoparticles [23, 24]. Organic PAs, such as chlorin e6 (Ce6) and methylene blue (MB), have limited water solubility and photostability, while metal or semiconductor oxides suffer from poor biocompatibility and are hard to be cleared through the renal system, leading to the risk for visceral deposition of heavy metal elements [25]. To overcome these obstacles [26], carbon nanomaterials (CNMs) with their good biocompatibility [27], deep tissue diffusibility [28] and strong optical absorption characteristics [29] have been demonstrated to be desirable for various phototherapeutic applications. Figure 2 summarizes the use of CNMs as PAs for phototherapy.

## 2 Carbon-based materials for phototherapy

Carbon is the fourth most abundant element in the Universe and Earth-abundant [30]. Carbon can also be found in all types of life. Historically, carbon materials were known to exist only in three forms: amorphous carbon, graphite, and diamond. In the last several decades, carbon research attracted two Nobel Prizes to recognize the discoveries of C_60_ and graphene, along with a Kavli Prize for the discovery of carbon nanotube [30, 31]. It was demonstrated that carbon materials can have remarkably different properties depending on how the carbon atoms are arranged and what are their molecular dimensions/shapes [32]. Therefore, carbon can provide enormous opportunities for developing new materials with novel properties for various applications. Owing to their unique physicochemical properties, good biocompatibility, large surface area, easy surface modification, earth-abundant and low-cost, carbon materials have attracted a great deal of interest for a wide range of potential applications [33, 34] including biomedical applications for drug delivery [35], tissue engineering [36], cancer diagnosis and therapy [37, 38], bio-sensors [36, 39, 40], Alzheimer’s disease [19, 41], biomedical imaging [42] and anti-bacteria [43].

Although a majority of pure carbon materials are inactive as phototherapy agents, several surface functionalization strategies have been developed to assist CNM PAs to show good performance in phototherapy [44]. The design of carbon-based materials as phototherapy agents can be divided into the following three categories:

(1) Reducing hydrophobicity to increase the water solubility; modification with:
  a. polymer: PEGylation (also extend the half-life of nanoparticles in blood circulation) [42]
  b. DNA-assisted dispersion [45]
(2) Targeting: e.g., improving tumour specificity and recognizing any remaining tumour cells at the site of distant metastases through modification with:
  a. aptamers (AS1411 [46], R13 against epidermal growth factor receptor [46])
  b. peptides: NGR (Asn-Gly-Arg) [47]
  c. proteins (transferrin and antibodies) [25]
  d. vitamins (such as folic acid and biotin) [48]
(3) Enhancing NIR photon response; modification with:
  a. NIR response particles: i.e., organic molecules (chlorin [48], methylene blue [49])
  b. upconversion nanoparticles (UCNP) that can convert near-infrared light into visible light) [50]: Yb3+/Er3+/Gd3+ [51]

In addition, some more specific modification methods have also been reported, for instance, to enhance the half-life of blood circulation [44] or anti-hypoxia for cancer treatment [52]. Furthermore, more than one modification methods are often applied simultaneously for optimal phototherapy effects [53].

### 2.1 Carbon nanotubes (CNTs)

Carbon nanotubes (CNTs) are one or more graphite sheets that are rolled up into tube forms to form single-walled CNTs (SWCNTs) or multi-walled CNTs (MWCNTs) [54]. SWCNTs are the first CNMs used in phototherapy [55]. Owing to their excellent absorption characteristics and photothermal conversion efficiencies in NIR-I (700–950 nm) and NIR-II (1000–1700 nm) regions [56], CNTs are often used for PTT.

Panchapakesan et al. [55] employed SWCNT as photothermal therapeutic agents and reported their inhibition capacity for breast cancer BT474 cell line in response to 800 nm laser (approx. 50–200 mW/cm^2^) [55]. In 2012, the intrinsic fluorescence of SWCNTs over the NIR-II region was utilized for *in vivo* imaging [57]. Later, SWCNTs were employed as *in vitro* PDT agents in 2014 [58]. Apart from SWCNTs, MWCNTs have also been used as *in vitro* PTT agents since 2009 [59]. However, the poor solubility, polydispersity and non-selective performance of CNTs in addition to biosafety concerns limited their practical applications [45, 60]. Therefore, several modification methods have been developed for CNTs. At the early stage, cancer-targeting molecules such as antibodies or specific receptors were combined with CNTs for *in vitro* cancer cell inhibition. For example, in 2007, Shao et al. [61] modified SWCNT with an IGF1 receptor and HER2 to achieve the selective attachment of the modified SWCNT to breast cancer cells. After 3 min exposure to 808 nm light (∼800 mW/cm^2^), the modified SWCNT demonstrated complete inhibition of cancer cells *in vitro* whereas the non-modified SWCNT showed only 20% inhabitation on MCF-7. In 2008, Chakravarty et al. [62] coupled SWCNTs with anti-CD25 mAb for phototherapy of PTT tumour cells *in vitro* with NIR light (808 nm, 5 W/cm^2^, 7 min). Wang et al. [59] reported in 2009 that MWCNTs conjugated with anti-GD2 were selectively internalized by neuroblastoma cells via GD2-mediated endocytosis but not PC12 cells with a poor GD2 expression. For neuroblastoma cells, cell death was only found within the NIR espoused region (808 nm, 0.6–6 W/cm^2^ 10 min + 6 W/cm^2^ 5 min) [59].

**Figure 3:**
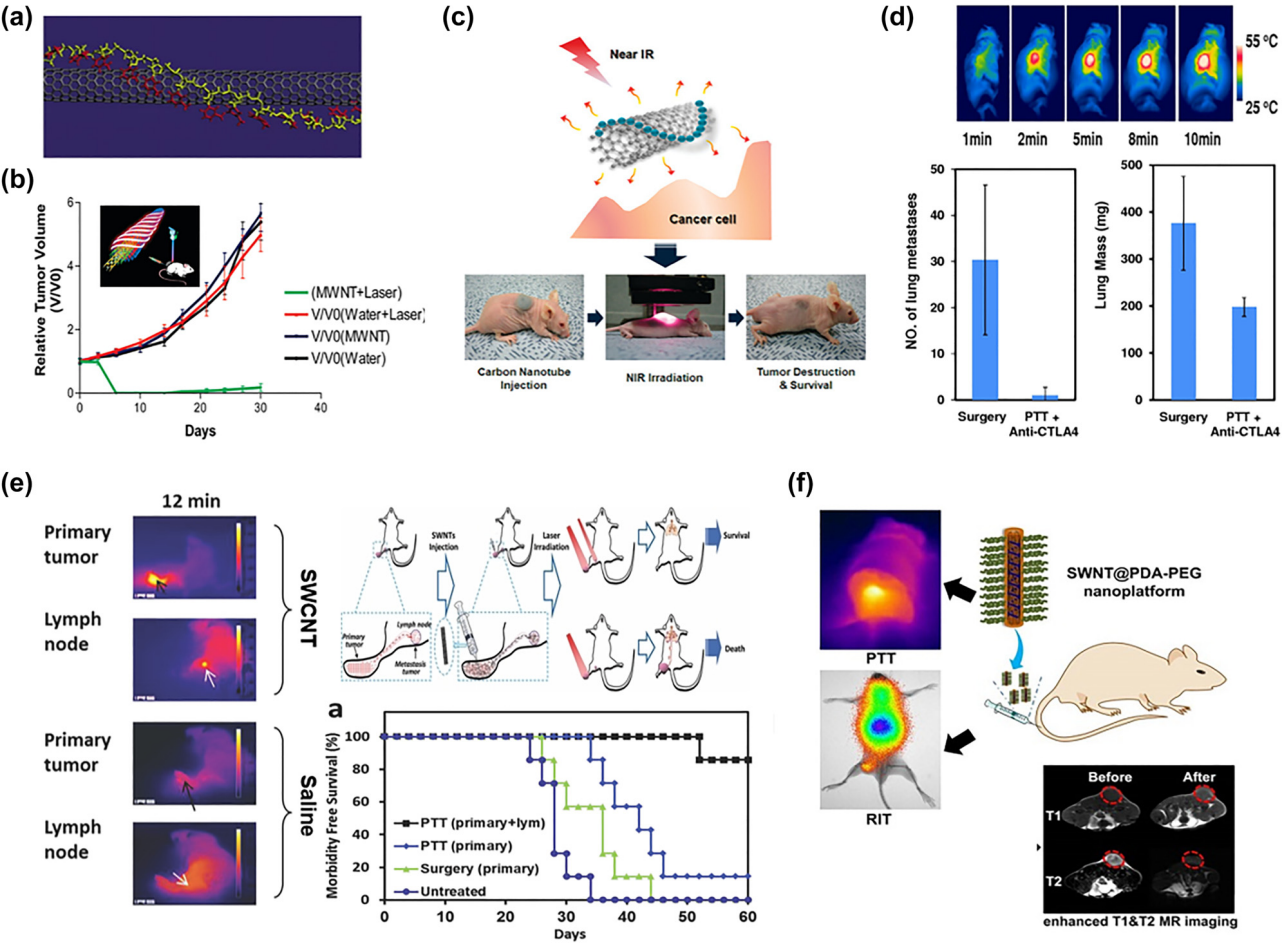
CNT as phototherapy agents: (a) DNA-assisted dispersion of CNTs [45], Copyright 2022 Nature Publishing Group. (b) DNA-attached MWCNTs for PTT [64], Copyright 2022 ACS AuthorChoice. (c) PEG-SWCNTs for NIR PTT [44], Copyright 2022 American Chemical Society. (d) immune responses triggered by anti-CTLA-4 SWCNT [71], Copyright 2022 John Wiley and Sons. (e) image guide-SWCNTs for PTT [72], Copyright 2022 John Wiley and Sons. (f) RIT&PTT by SWCNTs [73], Copyright 2022 Ivyspring International Publisher.

Hydrophobic compounds tend to have low bioavailability and have a tendency to be eliminated from the gastrointestinal tract [63]. The hydrophobic nucleobases of DNA could bind to CNT through hydrophobic-hydrophobic interaction and/or π–π stacking while the phosphodiester backbone of DNA can enhance the hydrophilicity of the DNA-attached CNTs (Figure 3a) [45, 64, 65]. Ghosh et al. reported the first intratumoural injection of DNA-attached MWCNTs as PTT agents to completely eliminate tumours in the PC3 tumour xenograft mouse model in response to NIR-II light (1064 nm, 2.5 W/cm^2^) (Figure 3b) [64]. Compared to the pristine MWCNTs, the DNA attachment increased the PTT heat production up to 2–3 times. MWCNTs have also been reported for chem-phototherapy by loading DOX, ICG and CD44 receptors for targeting hyaluronic acid (HA) [66]. However, it is still challenging for CNTs to reach the target area after intravenous injection. As demonstrated by intravenous and intratumoural injection mimicked models, intratumoural injection of PAs could achieve better results (100% inhibition of cancer cells) compared to intravenous injection of PAs (60% inhibition of cancer cells) [67]. However, intratumoural injection is facing the challenge of locating the tumour at the early stage and reaching the tumour site in the deep region. Nevertheless, several CNMs have been reported to be able to accumulate in the tumour tissues with/without targeting modification [25, 68], [69], [70].

PEGylation is commonly used to improve the solubility of nanoparticles and extend their half-life circulation times in bloodstream. In 2009, Moon et al. [44] reported PEGylated-SWCNTs for NIR PTT (808 nm, 76 W/cm^2^, 3 min) (Figure 3c). They found that PEGylated-SWCNTs could destroy tumour cells through partial skin damage in a noninvasive manner. It was revealed that there was no harmful side effect or tumour recurrence even after prolonged photothermal treatment for over 6 months [44]. Subsequently, Dai and coworkers [74] demonstrated the PEGylated SWCNTs for NIR light-induced (808 nm) PTT at a very low injection dose (70 μg of SWNT/mouse, 3.6 mg/kg, equivalent to 3.6 mg/kg) with a low laser irradiation power (0.6 W/cm^2^). After intravenous injection, PEG-SWCNTs showed a high elimination effect on tumours and their uptake could be imaged via the intrinsic NIR-photoluminescence (PL) from SWCNTs [74]. Liu and coworkers [71] reported that the combination of SWCNT with PEG and anti-CTLA-4 antibody for triggering an immunological response (Figure 3d) can not only photothermally ablate primary tumours but also significantly reduce the development of tumour metastasis in comparison to surgery (Figure 3d, the average number of metastasis sites of surgery: ∼30 per mouse, SWNT-based PTT plus anti-CTLA4 therapy: ∼1 per mouse). Apart from the excellent photothermal performance, CNTs can also act as PDT agents. In 2014, Zhou et al. [75] reported SWCNTs for photodynamic therapy by functionalizing SWCNTs with PL-PEG, which could be found in mitochondria in both normal and cancer cells or lysosomes macrophages depending on internalized methods, mitochondrial transmembrane and/or endocytosed. By further modifying SWCNT-PL-PEG with folate acid (FA), FA-SWCNTs could selectively enter the FR-positive cells to produce significant ROS under 980 nm laser(0.75 W/cm^2^, 2 min), inducing mitochondrial damage and consequent apoptosis [75]. The first SWCNT-based *in vivo* PDT study was reported by Wang et al. in 2014 using visible light (300 W lamp at 20 cm distance for 1 or 2 h). In this case, SWCNTs were covalently functionalized with PEI to demonstrate a promising anti-cancer effect via PDT *in vitro* and *in vivo* [58].

SWCNTs have also been applied to synergistic therapies, including PTT-CT (DOX [76], SNX-2122 [77]), imaging-guided PTT [72], PTT enhanced PDT [78] and PTT-RIT [73]. It has been reported that many aromatic drugs (e.g., DOX) could be loaded on CNTs via hydrophobic interaction and/or π–π stacking [76] to improve cellular uptake of the nano-carriers and for a more controllable drug release. In this context, Liu and coworkers synthesized PEG-modified mesoporous silica (MS) coated SWCNTs for cancer therapy with a synergistic effect by photothermally releasing anti-cancer drug DOX with NIR stimulation, leading to an outstanding synergistic tumour suppression at a low SWNT@MS-PEG dose (10 mg/kg and 4.3 mg/kg) and low laser power density (808 nm, 0.7 W/cm^2^, 5 min) [79]. In 2014, Liang et al. further reported the image-guided PTT for the effective destruction of primary tumours and cancer cells in sentinel lymph nodes, leading to a significant improvement in survival rate and metastasis inhibition (Figure 3e) [72]_._ In another study, Liu and coworkers combined PEG-SWNTs with a self-polymerized PDA shell, which could chelate Mn^2+^ for MRI imaging and enable efficient radioisotope labeling with 131I to allow for radioisotope therapy (RIT) to be combined with PTT (Figure 3f) [73].

To make multifunctional CNTs, they have been conjugated with multiple surface modifiers as imaging-guided triple-modal therapeutic (PTT/PDT/CT) agents for cancer treatments under 808 nm irradiation [80]. In this regard, Liang et al. modified CNTs with PDA and dopamine coating to achieve good solubility in water and low cytotoxicity. Certain CNT composites also showed excellent potential for bacteria-infected skin wound healing [81]. In summary, CNTs with strong NIR absorbance and high surface modification potential have been widely used in photothermal therapy and are exploited for photodynamic and other treatments recently. Furthermore, CNT-based multifunctional nanocomposites have been used for improved anticancer treatments with multiple synergistic therapeutic effects. Although research progresses achieved to date have demonstrated no *in vivo* adverse effects even over 6 months after PTT [44], the potential long-term cytotoxicity still requires further study [60].

### 2.2 Graphene and related materials

Graphene oxides (GO) have been used for photothermal therapy [43, 82–84]. The poor water solubility of graphene-based materials has limited their applications for phototherapy, though they have excellent NIR absorption. Nevertheless, good water solubility and stability have been achieved by conjugating with hydrophilic polymers, such as polyethylene glycol (PEG), via the surface carboxyl groups of GO [85, 86]. Having the graphitic sp^2^ arrangement, GO can also be functionalized through π–π stacking. Using both covalent and noncovalent bonding strategies [28] various aromatic drugs, including DOX [67, 87], SN38 [82] and camptothecin (CPT) [83], have been successfully attached to GO.

**Figure 4:**
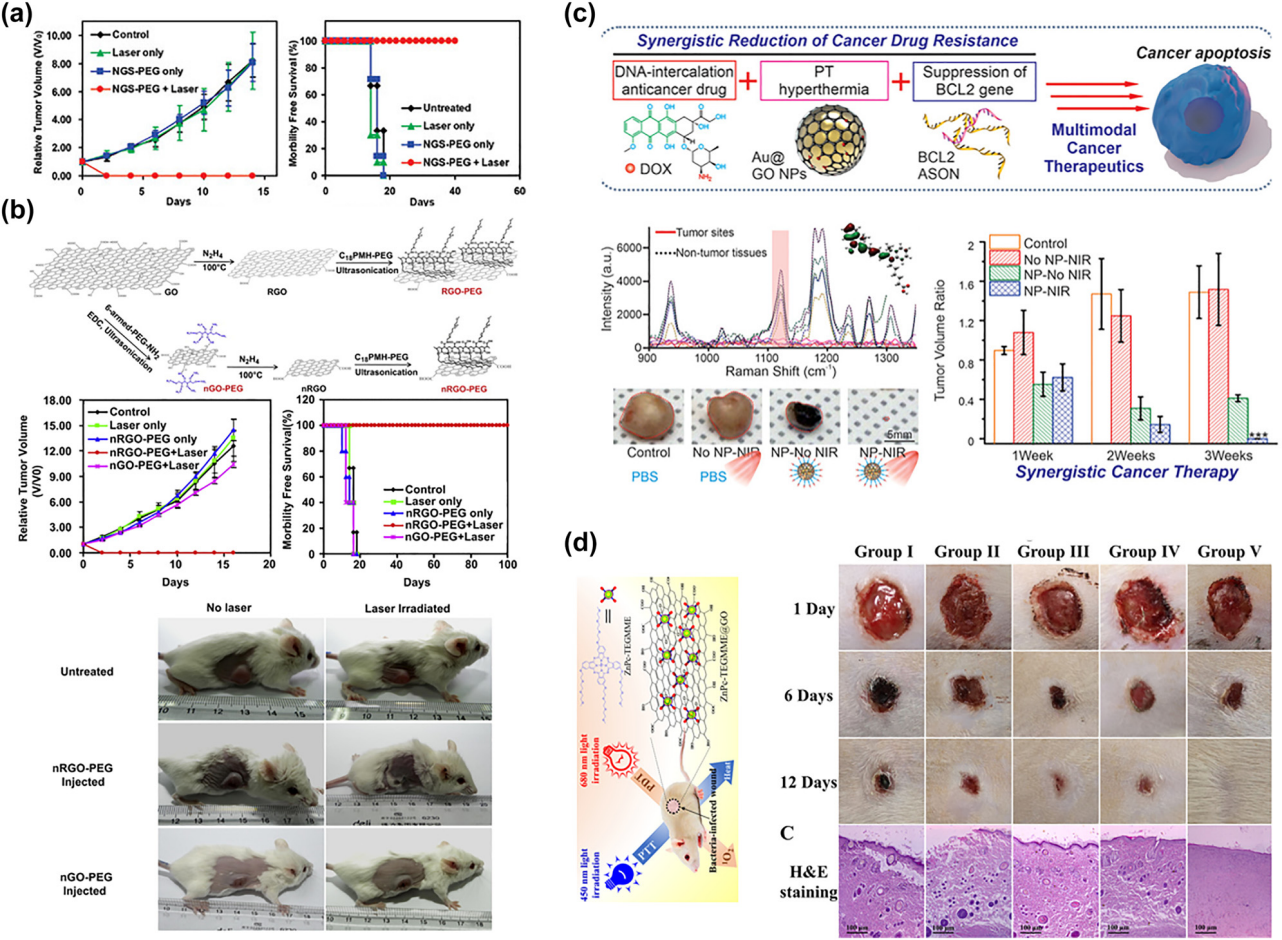
Graphene-based material for phototherapy: (a) PEG-nGO for PTT [84], Copyright 2022 American Chemical Society (b) rGO for PTT at low laser power [88], Copyright 2022 Elsevier publishing group. (c) PTT and CT ynergistic GO [67], Copyright 2022 John Wiley and Sons. (d) PTT&PDT GO for anti-bacterial [86], Copyright 2022 American Chemical Society.

In 2010, Liu and coworkers used PEGylated nanographene oxides (nGO) as an *in vivo* PTT reagent for 4T1 tumour-bearing mice in response to 808 nm laser (2 W/cm^2^, 5 min) [84]. It was found that PEG-nGO (GO dose: 200 μL, 2 mg/mL) showed high passive accumulation due to the enhanced permeability and retention in tumours and that the surface temperature could reach up to 50 °C upon irradiation versus only around 2 °C increase for mice without the injection of PEG-nGO. All tumours of the mice treated with PEG-nGO disappeared within 1 day after irradiation with no tumour regrowth over 40 days (Figure 4a).

GO, in a highly oxidized form, may have insufficient NIR absorption, leading to a low PTT conversion efficiency [89]. In 2011, Dai and coworkers proposed that reduced graphene oxides (rGO) could be a better candidate for PTT at a low dose and power intensity [89]. They proved that PEG-nano-rGO further modified with the peptide RGD (Arg-Gly-Asp) has a highly selective cellular uptake for effective photothermal inhabitation of U87MG cancer cells *in vitro* [89]. In 2012, Liu and coworkers reported that PEG-functionalized rGO could achieve excellent tumour elimination through intravenous injection upon exposure to ultra-low power intensity (0.15 W/cm^2^) 808 nm laser for only 5 min [88]. The long-term post-treatment effects were tracked for 100 days with no obvious side effects or death (Figure 4b). These results demonstrate a promising future for graphene-based PTT therapeutic approaches against cancer even at power intensity lower than that required by clinic regulation to avoid skin damage (0.38 W/cm^2^). While PEG-GO was mainly found in the liver, kidney and lung, PEG-rGO was found in the liver and spleen. Moreover, PEG-rGO had a longer phase blood circulation half-life of 17.5 h compared to 5.8 ± 2.8 h for PEG-GO [88]. In 2016, Chen and coworkers coated PEG-rGO with gold superparticles (PEG-rGO-GSPs) for enhanced PTT. It was found that tumours in mice intravenously injected with PEG-rGO-GSPs (200 μL, 1 mg/mL) were successfully eliminated without reoccurrence after irradiation with 808 nm NIR laser (0.8 W/cm^2^, the surface temperature reached 58 °C within 5 min) while rGo or laser alone showed a rapid tumour regrowth rate similar to the control group [90].

Graphene-based materials incorporated with other particles can be combined with other therapeutic approaches, including chemotherapy (DOX [67, 87]), RT [91], immunotherapy [92], gene therapy [93], PTT enhanced PDT [7], and *in vivo* imaging-guided PTT/CT [94]. For example, Zhang et al. [87] developed PEGylated-GO loaded with DOX to achieve combined chemo-thermal cancer therapy. PEG-GO-DOX with a pH-dependent drug release showed a favorable drug delivery to acidic TME. Compared with CT or PTT alone, a higher efficacy was obtained for PEG-GO-DOX under NIR light (808 nm, 2 W/cm^2^) with a lower side effect over DOX alone both* in vitro* and *in vivo* [87]. In 2015, Chen and coworkers combined rGO with DOX and gold nanorods to achieve the combined chemo-thermal tumour inhibition and controlled DOX release (808 nm, 0.5 W/cm^2^, 5 min). In this case, the *in vitro* half-maximal inhibitory concentration (IC50) of rGO-Au-DOX was reported to be 0.63 μg/mL; approximately 2 times lower than that of free DOX (∼1.34 μg/mL) in U87MG cells. For mice treated with the rGO composite by 808 nm laser at 0.5 W/cm^2^, tumour growth was completely inhibited with no reoccurrence for 14 days and survived over 40 days after the irradiation; showing a similar tumour growth rate and survival period to control mice [95]. Targeting molecules could also be conjugated onto GO to achieve selective chemo-PTT therapeutic effects. In 2019, Huang et al. reported *in vivo* chemo-PTT effects with LA-DOX-GO in response to a 660 nm laser [96]. By further combining DOX-GO and Au@GO-supported BCL2 for tumour specifically targeting via π–π stacking, accurate diagnosis and efficient therapeutics could be achieved simultaneously. After the intratumoural injection of GO composites, tumour cells showed a higher expression of BCL2 *in vivo* and suppression of the prosurvival gene, leading to a synergistic PTT and CT effect compared with the control groups (Figure 4c) [67].

Apart from being combined with CT, several PDT photosensitizers, including FDA-approved drugs like Ce6 [7, 97], indocyanine green (ICG) [98, 99], methylene blue [100, 101] and AIE [102], have also been conjugated to GO to achieve optimal phototherapeutic effects. In 2011, Liu et al. functionalized GO with PEG and Ce6 to demonstrate PTT-enhanced PDT against cancer cells in response to a 660 nm laser at low power (50 mW/cm^2^, 5 min). Gulzar et al. reported that synergic PTT and PDT therapeutic effects could be achieved *in vivo* under 808 nm light by combining UCNP with Ce6 co-conjugated GO [103]. They pre-irradiated PEG-Ce6-GO with 808 nm laser (0.33 W/cm^2^, 20 min), with which Ce6 has no absorption, but the absorption of GO can generate heat to induce a 5–6 °C mild temperature increase, increasing the cellular uptake of PEG-Ce6-GO composite to enhance the *in vitro* photodynamic therapy of cancer cells [7]. In 2019, Zhang et al. loaded wedelolactone (WED) and indocyanine green (ICG) on the GO through π–π stacking interactions, and found that the resultant GO-ICG-WED reached a high heating rate of 79 °C in 10 min with a high singlet oxygen generation rate under 808 nm irradiation [98]. It has been argued that GO acts as a highly efficient fluorescence quencher to dramatically reduce ROS generation even conjugated with PDT agents [104]; however, only slight singlet oxygen generation quenching was observed for the GO-C_60_ hybrid [105]. In 2018, Sun, et al. [102] incorporated AIE (aggregation-induced emission) molecules (i.e., TPE-red) into PEG-GO and achieved bioimaging in targeted cells and mouse ear blood vessels. They demonstrated that the TPE-PEG-GO hybrid increased the significance of ROS production under 450 nm laser in PBS solution with significantly increased *in vivo* therapeutic effects on tumour inhibition over control groups [102].

Graphene-based materials were also applied for photoactive antimicrobial effects [9] and Alzheimer’s disease regulation [19]. Since 2010, graphene-based materials have been reported to show *in vitro* bacteria toxicity through membrane damage [8] caused by its sharp edges and phospholipids extraction between graphene and lipid molecules [106]. Thus, graphene-PTT conjugation has also been developed for anti-bacterial therapy. In 2017, tobramycin (Tob, a wide spectrum antibiotic molecule) and Cu doped GO hybrids were reported for *in vitro* and *in vivo* NIR (980 nm, 1.5 W/cm^2^, 5 min) PTT&PDT for antibacterial infection therapy with up to 70% biofilm eradication and nearly 100% bacteria inhibition [9]. Although CNMs doped with heavy metal ions could enhance PTT and PDT against bacterial infection, the release of Cu or other heavy metals in the human body often causes toxicity [86]. In 2021, Mei et al. [86] combined PEGylated GO with ZnPc for triple antibacterial therapy through dual irradiation (450 nm PTT and 680 nm PDT) for 10 min to generate hyperthermia of nearly 100 °C at the surface and singlet oxygen to oxide the bacterial membrane, achieving a synergistic antibacterial efficiency (for both gram-positive and gram-negative) *in vitro* and *in vivo*. For the groups treated with the GO hybrid, no bacteria residues on the surfaces of the rat wounds were observed 12 days after irradiation (Figure 4d) [86]. Furthermore, Liang et al. encapsulated GO with hydrogel for drug-resistant bacteria-infected wound healing [107]. Phototherapy is a promising strategy for Alzheimer’s disease (AD) via −β protein (Aβ) regulation, which is a major pathological hallmark of AD. Both PDT and PTT from CNMs can be applied to mitigate abnormal self-assembly Aβ-induced neurotoxicity [19]. In 2012, Li et al. modified GO with thioflavin-S to generate heat to dissociate the Aβ fibrils under NIR laser irradiation (1 W/cm^2^, 8 min) in a mice cerebrospinal fluid [41].

### 2.3 Fullerene (C_60_)

Just like CNTs and graphene-based materials discussed above, fullerene C_60_ has also been widely used for PTT/PDT. Having a soccer-ball-like fully-conjugated carbon structure consisting of 12 pentagons and 20 hexagons facing symmetrically, fullerene (C_60_) can strongly absorb visible light with interesting photoexcitation properties upon light exposure [108]. Upon irradiation, C_60_ is excited to a triplet state, which can transfer its energy to surrounding oxygen to form singlet oxygen (^1^O_2_) [105]. However, the poor NIR absorption and insolubility in the biological solution of unmodified C_60_ have strongly limited C_60_ for PDT [109].

UV–vis excitation of C_60_ has been applied for anti-bacteria under visible light and fullerenes could also be used in PTT, particularly modified C_60_ is already used for photodynamic cancer therapy [110]. Since 1997, C_60_ has been reported as the PDT agent for ^1^O_2_ formation under visible light for virus inactivation in biological fluids (Semliki Forest virus (SFV, *Togaviridae*) and vesicular stomatitis virus (VSV, *Rhabdoviridae*)) [111]. In 2003, Yamakoshi et al. reported C_60_ for ROS generation in aqueous media under visible light and demonstrated a DNA cleavage capacity in DNA extracts [112]. To overcome the insolubility limitation of C_60_ mentioned above, serval modification strategies by introducing hydrophilic moieties, including water-soluble trimalonic acid functional groups like N-methylpyrrolidinium (BB4) [113], PEG [114], and block copolymers micelles, have been reported to acquire hydrophilic properties [115]. For instance, Hamblin and coworkers [115] reported cationic fullerenes as antimicrobial phototherapy agents; they modified C_60_ with pyrrolidinium groups, such as BF1–3 (three polar diserinol groups) and BF4–6 (a second series with one, two, or three quarternary pyrrolidinium groups), for anti-bacteria PDT under visible light irradiation (400–700 nm, 200 mW/cm^2^, 5 min). It was found that cationic C_60_ showed rapid and broad killing effects on more than 99.99% of bacterial and fungal cells and showed better selectivity than that widely used antimicrobial PDT agents (e.g., TBO, toluidine blue O) toward mouse fibroblast cells (L929) [115]. Later in 2011, Hamblin and coworkers used the same cationic fullerenes for intraperitoneal (IP) carcinomatosis PDT in the mice model (colon adenocarcinoma cell line (CT26) bearing BALB/c mice, IP injection) under visible light (400–700 nm) [113]. Krishna et al. [110] reported that up to 72% tumour size reduction under 785 nm laser irradiation of a water-soluble polyhydroxy fullerene (HPF) for 10 min (500 mW/cm^2^). Besides, HPF could also be used to image tumours with photoacoustic tomography (PAT) in BT474-bearing mice after intratumoural injection.

Although water solubility was improved, the phototherapy of modified fullerene was still limited to intratumoural injection. To achieve *in vivo* targeted area delivery of C_60_, PEG conjugated C_60_ has been studied for PDT of cancer *in vivo* under visible light [116]. PEG-modified C_60_ could be cooperated with other particles to achieve multifunctional systems. For example, in 2007, Liu et al. chelated Gd^3+^ to C_60_-PEG to enhance PDT efficacy with MRI under visible light (400–700 nm, 89.2 mW/cm^2^, 10 min), devising a therapeutic and diagnostic hybrid system in tumour-bearing mice (AR1/CDF1 mice) [114]. After intravenously injecting C_60_-PEG-Gd into mice, a sufficiently strong MRI signal from the tumour tissue was obtained for clinical MRI diagnosis.

Since C_60_-based PDT shows no specificity to tumour cells, tumour targeting agents, including peptides [47], aptamers [46], antibodies, and specific receptor-related molecules, were applied to further improve selectivity for selective inhibition of the tumour growth. In conjugation with tumour-targeting ligands (e.g., NGR that could cognize the CD13 isoform and tumour vascular), C_60_ showed a specific PDT inhibition of MCF-7 cells [47]. In 2010, Liu & Tabata functionalized C_60_ with pullulan (a polysaccharide with high affinity for asialoglycoprotein receptors; highly expressed at hepatocytes surface) for PDT of a HepG2 hepatoma cell line with high selectivity, leading to a 60% death of HepG2 cancer cells 6 h [117].

C_60_ could also be doped with other anti-cancer drugs (DOX [118]), metals [119], other CNMs or PAs (e.g., Ce6) [53] for CT-PDT and PTT-PDT synergic therapies. Indeed, Shi et al. [119] conjugated C_60_ with PEG, iron oxides (IONPs), and PDT anti-cancer drug HMME for PDT. By combining with C_60_, HOMME showed a 23-fold higher uptake of the tumour over HMME with significantly stronger killing effects. The resultant C_60_ hybrid was found in the liver, spleen, kidney, and tumour 3 h post-injection and showed excellent *in vitro* and *in vivo* PDT efficacies under 532 nm laser irradiation (300 mW/cm^2^, 5 min) [119].

Another form of fullerenes family C_70_ has also been applied for phototherapy. In 2016, Guan et al. decorated Amphiphilic TF-C_70_ with OEG2-modified Ce6 via a coupling reaction between amine groups on OMG2-Ce6 and carboxylic groups on TF-C_70_ to produce a water-soluble TFC_70_-OEG2-Ce6 (FCNVs) with 57 wt% Ce6. FCNVs demonstrated an imaging-guided PDT under NIR light at low laser intensity (660 nm, 100 mW/cm^2^, 10 min) [53]. Tumours (4T1 bearing Balb/c) treated with FCNVs with irradiation became scabby and ablated after 7 days and 15 days, respectively, while saline groups treated with the light or FCNVs alone showed continuous tumour growth for 15 days. FCNVs were found to accumulate in the liver, kidney, and tumour within 1–4 h post-injection while a negligible amount was detected in the spleen, lung muscle and heart. The half-life time was reported to be 73.6 h in the tumour. Overall, FCNVs showed no obvious damage to major organs and could be excreted by the liver and kidney with long-time blood circulation [53].

PEG crosslinked C_60_ was reported to be able to photoactively ablate various malignant cells after multimeric modification. In this context, Lee et al. reported [120] that Ce6 and folate conjugated PEG-C_60_ (multimeric C_60_) showed promising PTT and PDT therapeutic effects toward KB cancer, but with arthritis, after in intravenous injection. The surface temperature was found to reach 44 °C with tremendous singlet oxygen generation under 670 nm laser (300 mW/cm^2^, 10 min), resulting in arthritic progress inhibition in the arthritis-induced DBA/1 J mice mode and significant tumour volume regression in KB tumour-bearing mice (10 mg/kg).

### 2.4 Carbon quantum dots (CQDs)

Carbon quantum dots (CQDs) refer to CNMs sizes less than 10 nm [121]. Because different synthesis methods and precursors are used, CQDs are also called carbon dots (CDs) or graphene quantum dots (GQDs) for the dots synthesized from graphene. To avoid unnecessary confusion, we refer to all of them as CQDs in this review. Owing to their small size, tunable fluorescence, excellent water solubility and biocompatibility, high thermostability and photostability, CQDs are widely used for bio-applications [122], including biosensing [123, 124], bioimaging [125], and drug delivery [126]. Due to their size-dependent optical and other properties, CQDs could be used in both PDT and PTT within different wavelength ranges. CQDs show excellent renal clearance within (24–72 h) and cause no significant organ damage or inflammation, though CQDs predominantly accumulated in kidneys and liver, then spleen and lung [25, 127], [128], [129], [130].

**Figure 5:**
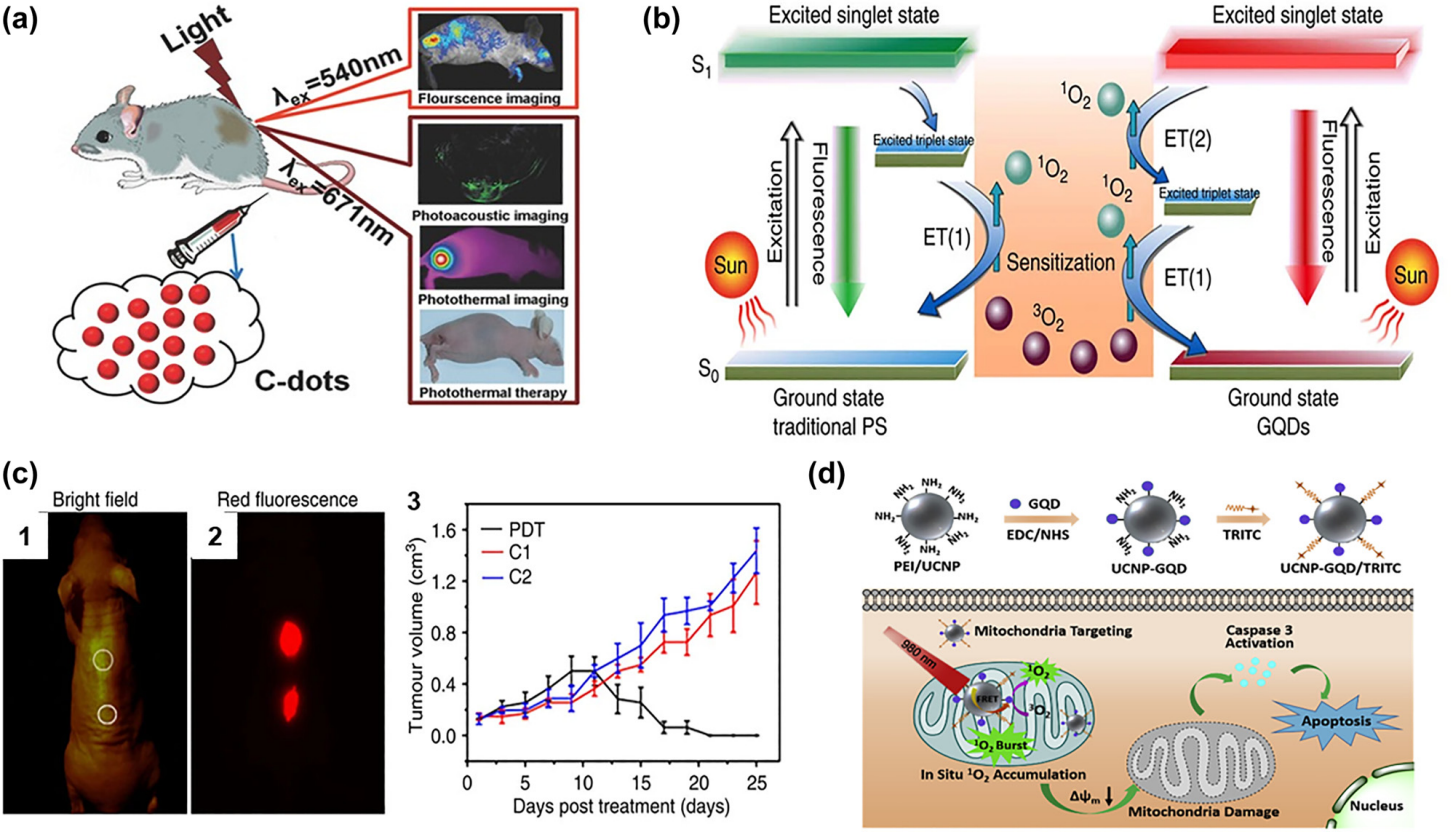
CQDs for phototherapy: (a) Red-emission CQDs for imaging and PTT, Copyright 2022 John Wiley and Sons. [130], (b & c) CQDs for PDT [133], Copyright 2022 Ge, J. et al. (d) CQDs for NIR-II mitochondria specific PDT [134], Copyright 2022 Elsevier publishing group.

Unlike other hydrophobic CNMs, hydrophilic CQDs could be directly applied for phototherapy. In 2015, Wang et al. reported sulfur-coated carbon quantum dots (S-CQDs) for PTT with a 38.5% photothermal conversion efficiency under a 671 nm laser at 2 W/cm^2^ (Figure 5a). S-CQDs accumulated in the tumour area after 2–5 h intravenous injection (2 mg/mL, 100 μL) and maintained photoacoustic imaging and fluorescence (*λ*_ex_ = 540 nm) signals for image-guided photothermal therapy toward Hela tumour-bearing mice. S-CQDs treated groups showed a significant temperature increase (>60 °C) at the tumour area, along with tumour size suppression for 18 days after 671 nm laser exposure for 10 min, while mice in the laser alone or S-CQDs in dark groups showed continuous tumour growth [130]. In 2017, Wang and coworkers doped CQD with Se to further improve the PTT efficiency up to 58.2% at 635 nm laser (2 W/cm^2^) [131]. In 2018, Bao et al. [25] reported a 59% photothermal efficiency for NIR-PTT (at a low laser intensity; 655 nm, 1 W cm^2^, 5 min) with photoacoustic imaging capacity for CQDs from the classical route involving citric acid and urea [132] without any further modification. CQDs were found to accumulate in the tumour site after intravenous injection (1 mg/mL, 200 µL) and the maximum accumulation was reached at 3 h post-injection. Upon irradiation, the temperature in the tumour area reached 59–71 °C for the CQDs-treated groups, leading to 100% tumour reduction in H22 tumour-bearing ICR mice after 14 days [25].

CQD can be used as a PDT reagent itself or doped with other PAs via covalent binding [135, 136] or supramolecular self-assembling (π–π stacking) [137]. In 2012, Markovic et al. reported the use of CQDs as photocatalysts for *in vitro* PDT cancer treatment under blue visible light (470 nm). After irradiation (470 nm, 1 W) for 10 min, the cell viability of human glioma cells U251 was decreased by singlet oxygen generated from CQDs in the treated groups. It was found that the CQD-induced cell death pathways include both apoptosis and autophagy-induced PDT [138]. In 2014, Wang and coworkers synthesized CQDs, which showed a broad absorption in the visible light region with an emission peak at 680 nm. These CQDs exhibited a high ^1^O_2_ generation yield for efficient tumour inhibition under white light (400–800 nm, 80 mW/cm^2^, 10 min) in the MDA-MB-231 bearing mice model (Figure 5b). Tumours in irradiated groups treated with the CQDs showed a tumour size decrease after 9 days while tumours treated with CQDs or light alone continued to grow for 25 days (>1 cm^3^) (Figure 5c) [133]. Due to the poor tissue penetration of visible light, however, this application is limited to skin cancer. To overcome the drawback of tissue penetration and potential damage under visible light, several efforts have been made to push CQDs toward the NIR region. Zhao et al. prepared N, P co-doped red-emission CQDs (NPR-CDs) with a ^1^O_2_-generation capacity in response to visible light (532 nm). NPR-CQDs showed PDT efficiency toward A549 cancer cells and tumours bearing mice (2 mg/kg, irradiation 2 h post-intratumourally injection) under 532 nm laser (100 mW/cm^2^) for 4 min [139]. In 2017, Li et al. [140] synthesized near-NIR responsive N-CQDs from hydroxylphenyl triphenyl porphyrin (TPP) and chitosan via a hydrothermal method. N-CQDs thus produced showed phototherapeutic effects (1 h after intratumoural injection, 625 nm 16 m W/cm^2^, 1 h) to reduce tumour volume from 100 to 56 mm^3^ in mice while the control group reached ∼800 mm^3^. Although TPP itself showed a 52.74% *in vivo* tumour inhibition, the N-CQDs generated from TPP exhibited a much higher inhibition rate of 82.84% [140]. On the other hand, intravenous injection of near-NIR-responsive self-assembly of PEG-coated CQDs demonstrated an efficient singlet oxygen generation with a quantum yield of 45.4% in water or 34% in PBS under the 671 nm irradiation. However, the NIR-light-responsive PDT cytotoxicity reached up to ∼99% at 200 μg/mL of 4T1 cells (671 nm, 60 mW/cm^2^, 10 min). CQDs were accumulated in the tumour within 8 h post-i.v.-injection with excellent PDT effects after irradiation, leading to a significant reduction in tumour volume [70].

Most PAs require additional modification to obtain nucleolus-targeting ability, while some CQDs demonstrate the intrinsic nucleolus-targeting capability that could selectively label subcellular organelles [141–143]. For example, Pang et al. [143] developed nucleolus-targeting CQDs with enhanced light-induced photocytotoxicity (400–700 nm, 0.1 W/cm^2^, 20 min). The synergy between targeting and photodynamic therapy has been exploited in both *in vitro* and *in vivo* for more effective treatment.

Due to their excellent water solubility, modified CQDs could also act as a carrier for water-insoluble PAs (e.g., Ce6) [144]. As early as 2012, Chen and coworkers [144] decorated CQDs with Ce6 and PEG via covalent binding and demonstrated a promising anti-cancer PDT effect induced by CQDs-Ce6 at low laser intensity (671 nm, 100 mW/cm^2^, 10 min). Specifically, they found that CQDs-Ce6 accumulated in the tumour region after 2–4 h injection with an excellent tumour inhibition ability under irradiation whereas dark groups or Ce6 alone under irradiation showed no significant tumour volume growth compared to the saline control groups [144]. In 2016, Du et al. [69] reported that Ce6 could also be loaded onto CQDs via a disulfide bond, stabilized with PF-12 (CQD–SS–Ce6), and released through cleavage of the disulfide linker under the tumour intracellular GSH condition. CQDs injected via the vein (Ce6: 2.5 mg/kg) were found to accumulate in the tumour and liver after 30 min irradiation (650 nm, 200 mW/cm^2^, 30 min), leading to over 80% tumour reduction in Hela-bearing mice after 14 days.

The application of CQDs for phototherapy, however, is still limited by the potential tissue damage under high PTT power intensity (2 W/cm^2^) and the limited penetration depth for visible light. Several efforts have been reported to push CQDs toward the NIR region with a deeper tissue penetration. In 2016, Zheng et al. [145] synthesized NIR-emitting CQDs (from cyOH and PEG800) with a maximum absorption at 370 nm and photothermal conversion efficiency of 38.7% under an 808 nm laser (2 W/cm^2^). These CQDs demonstrated a high *in vitro* viability inhibition to HepG2 and CT26 cells (less than 13% and 25%, respectively) upon irradiation (2 W/cm^2^) for 5 min. The surface of CQDs-treated mice (CT26 tumour-bearing BALB/c) reached 45 °C after 5 min light exposure (808 nm, 1–1.5 W/cm^2^) and reached 91% tumour inhibitory rate on day 11 while the saline group and CQDs alone only showed 0% and 7.2% tumour inhibitory rate, respectively [145]. In 2018, Geng et al. [146] prepared N-CQDs from TNP and BPEI, and found a PTT efficiency of 38.3% in response to 808 nm laser at low power intensity (0.8 W/cm^2^, 5 min). At a low intratumoural injection dose (100 μg/mL, 100 μL), Hela-bearing balb/c nude mice showed 100% tumour destruction [146].

UCNPs that can convert NIR light into visible light have also been doped into CQDs for responding to NIR. In this context, CQDs that can efficiently produce ^1^O_2_ production under UV–vis light were doped with UCNP and TRITC for mitochondrial *in-situ* ^1^O_2_ PDT under 980 nm laser (0.5 W/cm^2^, 20 min) (Figure 5d) [134]. Zhang et al. reported that 4T1-bearing mice treated with UCNP-CQD and UCNP-CQD/TRITC with irradiation showed tumour inhibition rates of 70.2% and 75.3%, respectively, while tumour volume in control groups increased by about 11 folds after 20 days [134].

In addition to the CQDs responsive to the NIR-I discussed above, Liu et al. [68] reported the NIR-II responsive CQDs in 2020 (1064 nm, 0.4 W/cm^2^ for 24 min). The reported CQDs have a strong absorbance in the NIR-II region (∼1070 nm) and a photothermal efficiency of 33.45% under a 1064 nm laser (1 W/cm^2^). Both *in vitro* and *in vivo* PTT therapeutic effects were confirmed; CQDs-treated groups under irradiation showed significant tumour growth inhibition while the tumour sizes in control groups reached ∼6.2 times compared to their initial size after 14 days. The circulation half-life of CQDs in blood was reported to be 1.59 h. CQDs were found in the kidney, spleen, lung, tumour and heart 24 h after intravenous injection with no obvious toxicity to these organs, as evidenced by histological Analysis and gene expression analysis [68].

Photo-responsive CQDs could also be cooperated with other strategies, such as chemotherapy [147] and NO delivery [148], to achieve synergic cancer therapeutic effects. Moreover, apart from anticancer applications, CQDs can also be implied for photo-induced anti-bacteria and AD regulations [19]. For example, Ristic et al. reported CQDs synthesized via electrochemical methods showed PDT anti-bacterial effects against *Staphylococcus aureus* and *Escherichia coli* under visible light (470 nm, 1 W) [149]. Zhang et al. developed HA-conjugated CQDs that could generate singlet oxygen under 650 nm laser irradiation, showing a selective inhibition of CD44-overexpressing cancer cells [150].

### 2.5 Carbon nitride (C_3_N_4_)

Carbon nitride is a promising photocatalyst, but pure C_3_N_4_ absorb visible light only. This restricts its phototherapy applications due to the low penetration depth associated with and potential skin damage caused by visible light [151–153]. Nevertheless, metal-doped carbon nitride has been reported for NIR-PDT [154]. In 2019, Li, et al. [154] loaded Ru and Fe on HOP-conjugated C_3_N_4_ (FCRH) for O_2_ self-supplement PDT under two-photon light (800 nm, 2.7 W, 5 min). After accumulation in the tumour tissue, FCRH can overcome hypoxia via O_2_ generation from water splitting under irradiation and enhanced PDT ^1^O_2_ generation, leading to significant tumour inhibition for 14 days [154]. Also, Taheri et al. [155] reported the use of mesoporous g-C_3_N_4_ (mgp-C_3_N_4_) without any modification for both *in vitro* and *in vivo* for PDT under 490 nm light (50 W, 1 h) in 2020. C_3_N_4_ was found to accumulate in the liver, lung, spleen, and tumour site, after intravenous injection, with no obvious organ damage. Both intratumoural and intravenous administration of the C_3_N_4_ resulted in a significant decrease in tumour volume after light exposure [155].

C_3_N_4_ could also be doped with other anti-cancer drugs for synergic therapies. For example, C_3_N_4_ nanosphere (HCNS) could load DOX for PDT-enhanced CT under visible light [156]. In 2020, Sun et al. reported a sono-photodynamical therapy strategy based on C_3_N_4_ self-assembled with Ce6-incorporated polycaprolactone/gelatin (PG). The resultant C_3_N_4_ hybrids significantly boosted ROS generation through a synergistic 808 nm laser (1 W/cm^2^, 5 min) and 1 MHz ultrasound (1 W/cm^2^, 50% duty cycle, 5 min) excitation, achieving a 95.8% inactivation rate for breast cancer cells [157].

### 2.6 Other CNMs

In addition to carbon nanotubes, graphene, fullerene, and carbon quantum dots discussed above, many other carbon materials have also been studied for PTT applications. For instance, carbon shell combined with metal was reported for PTT-enhanced CT under 808 nm lasers for *in vitro* cancer therapy in 2021 [158]. Dai et al. reported that the intravenously administrated FeCo-coated graphitic carbon shell loaded with DOX (FeCo/GC-DOX) showed a 45% tumour regression in mice with 20 min light exposure (NIR, surface reached 43–45 °C) [159]. In 2019, Lin et al. reported carbon nanohorns (CNHs) doped with PC (PC-SWNH) for PTT and PDT combined cancer therapy under 650 nm laser (1 W/cm^2^, 10 min). SWCH was found to act as a promising PTT agent. Combined with PDT, PC-SWNH showed tumour inhibition both *in vitro* and vivo [160]. More recently, Chen et al., synthesized C_5_N_2_ for PDT in response to a 655 nm laser for O_2_ generation via water splitting, demonstrating a promising inhibition for hypoxic tumours [161]. In a somewhat related but independent study, Hu et al. designed a PDA conjunction with C_60_ and rGO, which caused significant apoptosis toward Hela cells under light irradiation (Xe light 400–1100 nm, 2 W/cm^2^, 9 min) through singlet oxygen generation from C_60_ in NIR region [109] without decreasing the PTT effect of GO. GO-C_60_ was further modified with hydrophilic PEG to enhance its solubility, and the resultant PEG-GO-C_60_ was used for combined PTT and PDT in an aqueous solution for cancer therapy and antibacterial therapy [109]. Apart from anti-cancer and anti-bacterial phototherapy, C_3_N_4_ and CQDs hybrids were reported for *in vitro* photoactive inhibition of Aβ aggregation by ROS generated under white light illumination [19].

Finally, nanodiamond (ND) as a new class of diamond-structured carbon nanomaterial at nanoscale (<10 nm) exhibits strong photo-responsive properties and easy surface modification potential, making NDs a competitive candidate for various bio-applications like drug delivery and anti-cancer phototherapy [162–164]. For example, Choi and coworkers [165] proposed the folic acid (FA) conjugated NDs for selective folate receptor positive cancer cells (KB) photothermal therapy. They reported a significant tumour volume reduction in KB-bearing mice after 14 days post intravenous injection and NIR irradiation (808 nm, 2 W/cm^2^, 5 min). Later in 2018, Choi and coworkers designed a PDT and PTT synergistic phototherapy based on NDs [166], by decorating the phase-change material (PCM) and Ce6 to the NDs.

In summary, Table 1 summarizes the CNMs for PTT/PDT cancer therapy. Similarly, CNMs can have a photo-active antimicrobial effect from both PTT and PDT effects. For example, Lu et al., functionalized fullerenes with cation photodynamic therapy for potentially deadly skin wounds with *ProteusMirabilis* infection. After white light exposure (400–700 nm, 180 J/cm^2^) fullerenes treated group showed 82% survival compared to 8% in the control groups [167]. In 2010 [8] Akhavan and Ghaderi reported PTT graphene-based materials for anti-bacterial therapy, which could also be doped with metal for PTT/PDT antibacterial infection [9]. Although heavy metal ions doped CNM could enhance PTT and PDT against bacterial infection, the release of Cu or other heavy metals could cause toxicity in the human body [86].

**Table 1:** Summary of CNMs for cancer treatments.

CNM	Application	NP components				Phototherapy				Year	Ref
		Target	PTT	PDT	Other	Wavelength	Power	Time	Therapeutic effects
						(nm)	(W/cm^2^)	(min)	
SWCNT	Anti-cancer PTT	–	SWCNT	–	–	800	∼50–200		*In vitro* breast cancer BT474 inhibition	2007	[55]
SWCNT	Anti-cancer PTT	–	SWCNT	–	–	808	1.4	2	*In vitro* cancer cells inhibition	2005	[168]
SWCNT	Anti-cancer PTT	IGF1 HER2	SWCNT	–	–	808	∼800	3	*In vitro* breast cancer cells MCF-7 inhibition	2007	[61]
SWCNT	Anti-cancer PTT	CD25 mAb	SWCNT	–	–	808	5	7	*In vitro* cancer cells inhibition	2008	[62]
MWCNT	Anti-cancer PTT	anti-GD2	MWCNT	–	–	808	0.6–6 + 6	10 + 5	*In vitro* cancer cells inhibition	2009	[59]
MWCNT	Anti-cancer PTT	–	MWCNT	–	DNA	1064	2.5	1.17	*In vivo* tumour disappeared irradiation 1.5 h after intertumoural injection	2009	[64]
MWCNT	Anti-cancer PTT + CT	CD44 HA	MWCNT	–	PEG DOX	808	1	5	In vivo tumour inhibition reduced cytotoxicity of DOX	2021	[66]
SWCNT	Anti-cancer PTT	–	SWCNT	–	PEG	808	76	3	*In vivo* intravenous injection tumour disappeared no recurrence over 6 months	2009	[44]
SWCNT	Anti-cancer PTT	–	SWCNT	–	PEG	808	0.6	5	*In vivo* low injection dose, 3.6 mg/kg intravenous injection tumour disappeared no recurrence ∼6 months	2010	[74]
SWCNT	Anti-cancer PTT	anti-CTLA-4	SWCNT	–	PEG	808	1	10	*In vivo* intravenous injection immune response tumour disappeared inhibited pulmonary metastasis	2014	[71]
SWCNT	Anti-cancer PDT	FA	–	SWCNT	PEG	980	0.75	2	*In vitro* FR-positive cells	2014	[75]
SWCNT	Anti-cancer PDT	–	–	SWCNT	PEI	300–2600	200 W at 20 cm		*In vivo* tumour inhibition	2014	[58]
							distance for 1 or 2 h				
SWCNT	Anti-cancer PTT image	–	SWCNT	–	PEG DOX MS	808	0.7	5	*In vivo* tumour inhibition	2015	[79]
SWCNT	Anti-cancer PTT + RIT	–	–	–	PEG Mn_2+_				*In vivo* tumour inhibition MRI imaging	2016	[73]
SWCNT	Anti-cancer PTT	–	SWCNT	–	PEG	808	0.5 & 0.8	12	Imaging destruction of primary tumours inhibited metastasis in sentinel lymph nodes	2014	[72]_._
CNT	Anti-bacterial wound healing PTT	–	CNT	–	QCS GMA	808	1.4	10	*In vivo* tumour inhibition noncompressible hemorrhage and wound healing	2018	[169]
SWCNT	Vascular inflammation PTT	–	SWCNT	–	Cy5.5 PEG	808	5	2	*In vivo* tumour inhibition tail injection	2012	[170]
SWCNT	*In vivo* tumour image	–	SWCNT	–	–	–	–	–	*In vivo* tumour inhibition imaging	2012	[57]
SWCNT-Fe_3_O_4_	PTT PDT CT	–	SWCNT	Fe_3_O_4_	DOX PEG	808	2	5	*In vivo* tumour inhibition	2018	[80]
GO	Anti-cancer PTT	–	GO	–	PEG	808	2	5	*In vivo* the tumour disappeared after 1 day, no recurrence	2010	[84]
rGO	Anti-cancer PTT	RGD	rGO	–	–	808	15.3	8	*In vitro* cancer cells U87MG inhibition	2011	[89]
rGO	Anti-cancer PTT	–	rGO	–	PEG	808	0.15	5	*In vivo tumour* elimination no obvious side effect after 100 days	2012	[88]
rGO	Anti-cancer PTT	–	rGO	–	PEG	808	0.8	5	*In vivo* tumour inhibition tumour elimination without reoccurrence	2016	[90]
GO	Anti-cancer PTT + CT	–	GO	–	DOX PEG	808	2	5	*In vivo* tumour inhibition PH-dependent drug release tumour elimination without reoccurrence	2011	[87]
rGO	Anti-cancer PTT + CT	–	rGO	–	PEG Au DOX	808	0.25	3	*In vivo* acid-triggered DOX release tumour growth inhibition	2015	[95]
GO	Anti-cancer PTT **+ CT**	LA	GO ICG(IR820)	–	DOX	660	1	5	*In vivo* pH-sensitive drug release tumour inhibition after 3 weeks	2019	[96]
GO	Anti-cancer PTT + CT	BCL2	GO	–	DOX Au	808	1.2	10	*In vivo* tumour inhibition	2021	[67]
GO Ce6 PEG	Anti-cancer PTT + PDT	–	GO	Ce6	PEG	808 + 660	0.33 + 0.05	20 + 5	*In vitro* cancer cells inhibition	2011	[7]
GO Ce6 UCNP	Anti-cancer PTT + PDT	–	GO	Ce6	PEG	808	0.72	10	*In vivo* tumour inhibition	2018	[103]
GO	Anti-cancer PTT **+ PDT**	–	GO	ICG WED	–	808	2	1	*In vivo* cancer cells inhibition tumour elimination after 14days	2019	[98]
GO	Anti-cancer PDT	–	–	AIE	–	450	0.2	5	*In vivo* tumour inhibition	2018	[102]
GO	Anti-cancer PTT-CT anti-cancer	–	GO	Fe	DOX PEG	808	1	5	*In vivo* tumour inhibition	2012	[94]
GO	PTT PDT	–	GO	MB	–	808	2	3	*In vivo* tumour inhibition	2013	[49]
GO	Anti-cancer PTT + PDT	Folic acid	GO	ICG	PEG	808	1.8	5	*In vivo* tumour inhibition	2021	[171]
C_60_	Anti-virus PDT	–	–	C_60_	–	–	–	–	*In vitro* virus inhibition	1997	[111]
C_60_ cationic	Anti-bacteria PDT	–	–	C_60_	BF1-6	400–700	120 J/cm^2^	5	*In vitro* more than 99.99% of bacterial and fungal cells	2005	[115]
C_60_ cationic	Anti-cancer PDT	–	–	C_60_	BB4	400–700	0.2	5	*In vivo* tumour inhibition intraperitoneal(IP) carcinomatosis	2011	[113]
C_60_ HPF	Anti-cancer PDT	–	–	C_60_	–	785	0.5	10	*In vivo* up to 72% tumour size reduction intratumourally injection	2010	[110]
C_60_	Anti-cancer PDT	–	–	C_60_	PEG Gd3+	400–700	0.089	10	*In vivo* tumour inhibition MRI imaging	2007	[114]
C_60_	Anti-cancer PDT	Pullulan	–	C_60_	–	400–700	8 W 2 cm	5	*In vitro* 60% death of HepG2 cancer cells	2010	[117]
C_60_	Anti-cancer PDT	–	–	C_60_	–	532	0.3	5	*In vivo* tumour inhibition	2013	[119]
C_60_	Anti-cancer and arthritis PDT	–	–	C_60_	–	670	0.3	10	*In vivo* tumour inhibition	2012	[120]
C_60_	Anti-bacteria PDT	–	–	C_60_	–	400–700	120 J/cm2	–	*In vitro* bacteria inhibition	2005	[115]
C_60_	Anti-cancer PDT	–	–	C_60_	–	400–700	8	5	*In vivo* tumour inhibition	2016	[53]
C_70_ OEG2 Ce6	Anti-cancer PDT	–	–	C_70_ Ce6	OMG2	660	0.1	10	In vivo tumour disappeared	2016	[53]
C_60_ IONPs HMME PEG	Anti-cancer PDT CT	–	–	C_60_ IONP	HMME PEG	532	0.3	5	*In vivo* tumour inhibition	2013	[119]
C_60_-GO	Anti-cancer PTT PDT	–	GO	C_60_	PDA	400–1100	2	9	*In vitro* cancer cell inhibition	2014	[105]
CQDs	Anti-cancer PDT	CQDs	–	CQDs	–	400–700	0.1	20	*In vivo* tumour inhibition	2020	[143]
CQDs Ce6 PEG	Anti-cancer PDT	–	–	Ce6	PEG	671	0.1	10	*In vivo* tumour inhibition	2012	[144]
CQDs	Anti-cancer PTT	–	–	CQDs	–	671	2	10	*In vivo* tumour inhibition	2015	[130]
CQDs	Anti-cancer PDT	–	–	CQDs	–	625	0.016	60	*In vivo* tumour inhibition	2016	[140]
CQDs	Anti-cancer PTT + PDT	–	CQDs	CQDs	–	800	0.5	10	*In vivo* tumour inhibition	2018	[172]
CQDs N, P co CQDs	Anti-cancer PDT	–	–	CQDs	–	532	0.1	4	*In vivo* tumour inhibition	2019	[139]
CQDs	Anti-cancer PDT imaging	–	–	CQDs	PEG	671	0.1	10	*In vivo* tumour inhibition	2019	[173]
CQDs	Anti-cancer PTT	–	–	CQDs	–	808	0.05	10	*In vivo* tumour inhibition	2019	[174]
C_3_N_4_ N-CQDs	Anti-cancer PTT + PDT	RGD	–	C_3_N_4_	PEG	980	1	5	*In vivo* tumour inhibition	2020	[52]
						630	0.155	5
CQDs	Anti-cancer PTT	–	CQDs	–	–	1064	0.4	24	*In vivo* tumour inhibition	2020	[68]
C_3_N_4_ GO Ce6	Anti-cancer PDT + Sono	–	GO	C_3_N_4_ Ce6	–	808	1	5	*In vivo* tumour inhibition	2020	[157]
ND	Anti-cancer PTT	FA	ND	–	–	808	2	5	*In vivo* tumour volume reduction	2016	[165]
ND	Anti-cancer PTT + PDT	–	ND	Ce6	PCM	670	2	4	*In vivo* tumour volume reduction	2018	[166]

## 3 Conclusion and outlook

As can be seen above, carbon nanomaterials exhibit good photo-responsive activities and stabilities to show great potential for phototherapy. Various functionalization strategies have been developed to enhance the low water-solubility, weak NIR absorption, and poor targeting capability intrinsically associated with certain specific CNMs for optimal PTT/PTD performance. Although significant progress has been achieved, the following issues still need to be addressed towards clinical translation.

Firstly, further study on long-term biosafety in the clinical use of CNMs is needed since carbon nanomaterials are very stable in biological environment. Although research progresses achieved to date have demonstrated no adverse effects in mice even over 6 months for some CNMs, their detailed metabolic behavior impacts, and long-term evaluations remain uncertain.

Secondly, to maximize the treatment effects on deep tumours/other diseases, CNMs needed to be combined with NIR-responsive PAs and/or other therapeutic strategies. However, most CNMs currently reported for PTT/PDT applications are responsive mainly to the NIR-I region, which still has some limitations on tissue penetration compared to the NIR-II region. So, more advanced PAs are needed to push the light absorption toward the NIR-II region, maximize the blood circulation time, and improve the conversion efficiency of light energy to chemical/thermal energy.

Lastly, more mechanistic studies are necessary to guide the design and synthesis advanced NIR-responsive CNMs with controlled PTT/PDT properties. This is the foundation for clinical translation and future personalized phototherapeutic treatment.

We believe that these issues and others will be resolved with the rapid development in carbon materials science and engineering, surface modification, catalytic medicine, and phototherapeutic technology. CNMs will revolutionarize the field of phototherapy and affect every aspect of our lives.

## List of Acronyms

AD: Alzheimer’s disease
AIE: aggregation-induced emission
BB4: N-methylpyrrolidinium
BF1–3: three polar diserinol groups
BF4–6: a second series with one, two, or three quarternary pyrrolidinium groups
C_60_
: fullerene
Ce6: chlorin e6
CNM: carbon nanomaterial
CNT: carbon nanotubes
C_3_N_4_
: carbon nitride
CPT: camptothecin
CQDs: carbon quantum dots
CT: chemotherapy
DOX: Doxorubicin
FA: folate acid
FCNVs: OMG2-Ce6 and carboxylic groups coupled on TFC70
GO: graphene oxides
GQDs: graphene quantum dots
HA: Hyaluronic acid
HOP: Poly (ethylene glycol) arms
HPF: water-soluble polyhydroxy fullerene
ICG: indocyanine green
IP: intraperitoneal
LA: lactobionic acid
MB: methylene blue
MWCNTs: multi-walled carbon nanotubes
MR: magnetic resonance
MS: mesoporous silica
NIR: near-infrared
NGR: Asn-Gly-Arg peptides
PA: phototherapeutic agents
PAT: photoacoustic tomography
PDA: polydopamine
PDT: photodynamical therapy
PEG: poly(ethylene glycol)
PEI: polyethylenimine
PL: photoluminescence
PTT: photothermal therapy
PT: phototherapy
PAs: phototherapeutic agents
RES: reticuloendothelial system
rGO: reduced graphene oxides
RIT: radioisotope therapy
ROS: reactive oxygen species
S-CDs: sulfur coated carbon quantum dots
SWCNT: single-walled carbon nanotubes
SFV: Semliki Forest virus
TBO: toluidine blue O
TME: tumour microenvironment
TPP: triphenyl porphyrin
UCNP: upconversion nanoparticles
VSV: vesicular stomatitis virus
WED: wedelolactone

## Supplementary Material

Supplementary Material Details
