# Supplementary material for: Carbon nanomaterials for phototherapy
Source: Nanophotonics. 2022 Nov 21;11(22):4955–76. doi: 10.1515/nanoph-2022-0574 (PMC11501915; doi:10.1515/nanoph-2022-0574)
Supplement: Supplementary file 1 — Supplementary Material Details [file j_nanoph-2022-0574_suppl.docx]

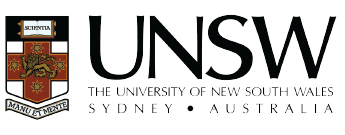
\

October 25, 2022

Drs.Tara Dorrian, Wubin Wen, and Ing, D. Couwenberg

*Nanophotonics*

Email:  [tdorrian@nanophotonics-journal.com](mailto:tdorrian@nanophotonics-journal.com)

Special Issue on *"*Novel two-dimensional materials based bio-nanophotonics*"*

Dear Tara et al.,

Many thanks for your email of October 13, 2021, in which you kindly sent us the Reviewers’ reports on our article entitled: “Carbon nanomaterials for phototherapy (NANOPH-2022-0574-R1)”. We have studied the Reviewers’ comments carefully. I am attaching a revised manuscript that incorporates amendments made in accordance with the Reviewers’ recommendations, along with our point-to-pint responses.

As you can see, we have clarified all the Editor’s and Reviewers’ concerns, which are very helpful to further improve the quality of our manuscript. For your convenience, we have highlighted the changes in our revised manuscript - please see: NANOPH-2022-0574-R1 (TrackedChanges). As you can see, the changes are very minor, and the revised manuscript is in an excellent shape for publication – please see: NANOPH-2022-0574-R1 (CleanCopy). I hope you will find our revision satisfactory to meet the standards of *Nanophotonics* and the paper is now acceptable for publication. If there is any further information you may require, however, please do not hesitate to let me know. Once again, thank you very much for your kind consideration and for all you and your colleagues have kindly done with our manuscript!

I look forward to hearing from you again.


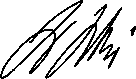


Yours sincerely,

Liming Dai

____________________________________________________________

Liming Dai, *PhD, FNAI, FAIMBE, FRSC, FEurASc*

Australian Laureate Fellow

Scientia Professor, SHARP Professor

Director of the Australian Carbon Materials Centre (A-CMC)

School of Chemical Engineering

University of New South Wales, Sydney, NSW2052, Australia

Associate Editor, *Nano Energy*

Phone: (02) 938-57544 (O); Email: [l.dai@unsw.edu.au](mailto:l.dai@unsw.edu.au)

<https://research.unsw.edu.au/people/scientia-professor-liming-dai>

https://www.acmc.unsw.edu.au/
